# Extracorporeal Membrane Oxygenation in Immunocompromised Patients With Acute Respiratory Distress Syndrome—A Retrospective Cohort Study

**DOI:** 10.3389/fmed.2021.755147

**Published:** 2021-12-03

**Authors:** Chiao-Feng Cheng, You-Yi Chen, Ming-Chieh Shih, Yi-Min Huang, Li-Jung Tseng, Chien-Heng Lai, Ting-Yuan Lan, Cheng-Hsun Lu, Song-Chou Hsieh, Ko-Jen Li, Nai-Hsin Chi, Hsi-Yu Yu, Yih-Sharng Chen, Chih-Hsien Wang

**Affiliations:** ^1^Department of Internal Medicine, National Taiwan University Hospital Yun-Lin Branch, Yun-Lin County, Taiwan; ^2^Institute of Epidemiology and Preventive Medicine, College of Public Health, National Taiwan University, Taipei, Taiwan; ^3^Cardiovascular Surgery, Department of Surgery, National Taiwan University Hospital and College of Medicine, Taipei, Taiwan; ^4^Department of Internal Medicine, National Taiwan University Hospital Hsin-Chu Branch, Hsin-Chu, Taiwan; ^5^Department of Internal Medicine, National Taiwan University Hospital, Taipei, Taiwan

**Keywords:** ECMO, extracorporeal life support, pulmonary hemorrhage, aspiration, autoimmune diseases, malignancy

## Abstract

**Objective:** Although the negative impact of immunosuppression on survival in patients with acute respiratory distress syndrome (ARDS) treated by extracorporeal membrane oxygenation (ECMO) is well known, short-term outcomes such as successful weaning rate from ECMO and subgroups benefit most from ECMO remain to be determined. The aims of this study were (1) to identify the association between immunocompromised status and weaning from ECMO in patients of ARDS, and (2) to identify subgroups of immunocompromised patients who may benefit from ECMO.

**Methods:** This retrospective cohort study enrolled patients who received ECMO for ARDS from 2010 to 2020. Immunocompromised status was defined as having a hematological malignancy, active solid tumor, solid organ transplant, or autoimmune disease.

**Results:** This study enrolled 256 ARDS patients who received ECMO, of whom 68 were immunocompromised. The multivariable analysis showed that immunocompromised status was not independently associated with failure to wean from ECMO. In addition, the patients with an autoimmune disease (14/24, 58.3%) and organ transplantation (3/3, 100%) had a numerically higher weaning rate from ECMO than other immunocompromised patients. For causes of ARDS, most patients with pulmonary hemorrhage (6/8, 75%) and aspiration (5/9, 55.6%) could be weaned from ECMO, compared to only a few of the patients with interstitial lung disease (2/9, 22.2%) and sepsis (1/4, 25%).

**Conclusions:** Immunocompromised status was not an independent risk factor of failure to wean from ECMO in patients with ARDS. For patients with pulmonary hemorrhage and aspiration-related ARDS, ECMO may be beneficial as bridge therapy.

## Introduction

Extracorporeal membrane oxygenation (ECMO) is considered to be an alternative treatment or rescue therapy for patients with acute respiratory distress syndrome (ARDS) (1–3). However, whether immunocompromised patients benefit from this invasive but potentially life-saving therapy remains uncertain (4, 5). Rapid progress has been made in the development of effective treatments for immunosuppressive diseases in recent years (6, 7), and the long-term survival of these patients has also improved (8–12). Therefore, although the negative impact of immunosuppression on survival in patients with ARDS is well known (13–15), an increasing number of clinicians are initiating ECMO in immunocompromised patients (1).

Previous studies reported a 6-month survival rate of only 25–30% in immunocompromised patients with ARDS who received ECMO (4, 5). These studies used relatively long-term outcomes, such as 6-month survival or survival rate to discharge to evaluate the benefit of ECMO. However, to assess the potential benefit of ECMO as a bridge to effective treatment of the underlying disease, short-term outcomes such as successful weaning rate from ECMO may also be important in these patients. Moreover, the poor survival rate in these patients raises the importance of identifying subgroups of immunocompromised patients who may benefit most from ECMO (16).

Therefore, the objectives of this single-center retrospective study were (1) to identify whether immunocompromised status was an independent risk factor for weaning from ECMO, and (2) to identify potential subgroups of immunocompromised patients who may benefit from ECMO.

## Materials and Methods

### Study Design

This retrospective cohort study was conducted at a tertiary referral hospital in Taiwan. The study complied with the Declaration of Helsinki, and it was approved by the Institutional Review Board of our hospital (201002034R). In our hospital, more than 150 rounds of ECMO are performed annually. The equipment and standardized management of cases have been detailed previously (17, 18). The data of all patients who received ECMO were entered prospectively into our database and reported to the Extracorporeal Life Support Organization. In our institute, ECMO serves as a rescue therapy for severe ARDS. Patients with ARDS were included in this study if they were 20 years of age or older and underwent ECMO between January 1, 2010 and January 10, 2020. ARDS was diagnosed according to the Berlin definition (19). Patients were excluded if they were under 20 years of age or this was not the first time they had received ECMO. If the patient received two modes of ECMO in the same episode, the first mode was recorded.

### Data Collection

The data collected from the database in this study included demographics, underlying comorbidities, duration between respiratory failure and ECMO setup, and outcomes from the medical records. The Charlson comorbidity index and Acute Physiology and Chronic Health Evaluation II (Apache II score) were recorded according to the last data recorded before receiving ECMO (20, 21). Because both scores include items related to immunocompromised status as defined in this study, we modified the scores by removing items related to immunocompromised status. We reported both original scores and modified scores. Dynamic driving pressure was defined as the difference between peak inspiratory pressure and positive end-expiratory pressure. Mechanical power was calculated as previously proposed (22). Immunocompromised status was defined as patients with (1) a hematological malignancy, (2) an active solid tumor or having received specific antitumor treatment within the previous year, (3) solid organ transplant before receiving ECMO, and (4) an autoimmune disease which fulfilled the classification or diagnostic criteria. The inotropic score was calculated as 100 × epinephrine dose (μg/kg/min) + 100 × norepinephrine dose (μg/kg/min) + dopamine dose (μg/kg/min) + dobutamine dose (μg/kg/min) (23).

### Statistical Analysis

We first assessed differences between the immunocompromised and immunocompetent patients by comparing their demographic characteristics and clinical outcomes. The clinical outcomes of interest were ECMO weaning rate, ECMO duration, hospital length of stay, rate of survival to discharge, and 6-month survival after receiving ECMO. Continuous variables were summarized as median with interquartile range (IQR), and compared using the Mann–Whitney U test; categorical variables were presented as numbers with percentages, and compared using Fisher's exact test. Survival curves within 6 months were estimated using the Kaplan-Meier method. Fisher's exact test for and Kruskal-Wallis test were used to compare the clinical outcomes between patients with different immunocompromised causes.

To further identify risk factors for failure to wean from ECMO and address confounding, we also compared the characteristics of the patients grouped by the status of weaning from ECMO. With failure to wean from ECMO as the outcome, univariable logistic regression analysis was performed for each potential risk factor, and factors with *p* < 0.05 were entered into multivariable logistic regression analysis. Significance was set at a *p* value < 0.05. Bonferroni correction was used in cases of multiple comparisons. We used a multiple imputation method to manage missing values, with additional complete data analysis performed as sensitivity analysis. We used propensity score analysis to validate the finding that immunocompromised status was not significantly associated with ECMO weaning failure. Propensity scores were calculated via a logistic regression analysis using covariates associated with ECMO weaning failure, including sex, body mass index, interval between intubation and ECMO cannulation, modified Charlson comorbidity index, modified APACHE II score, inotropic score, prone positioning before ECMO, and inhaled nitric oxide use before ECMO. Patients who were immunocompromised and those who were not were matched 1:1, based on their propensity scores using nearest-neighbor matching with a caliper at 0.002. Rubin's Rules were applied to pool parameter estimates. Statistical analyses were performed using MedCalc Statistical Software version 19.5.3 (MedCalc Software bvba, Ostend, Belgium) and SPSS software version 25.0 (SPSS Inc, Armonk, NY).

## Results

**Figure 1:**
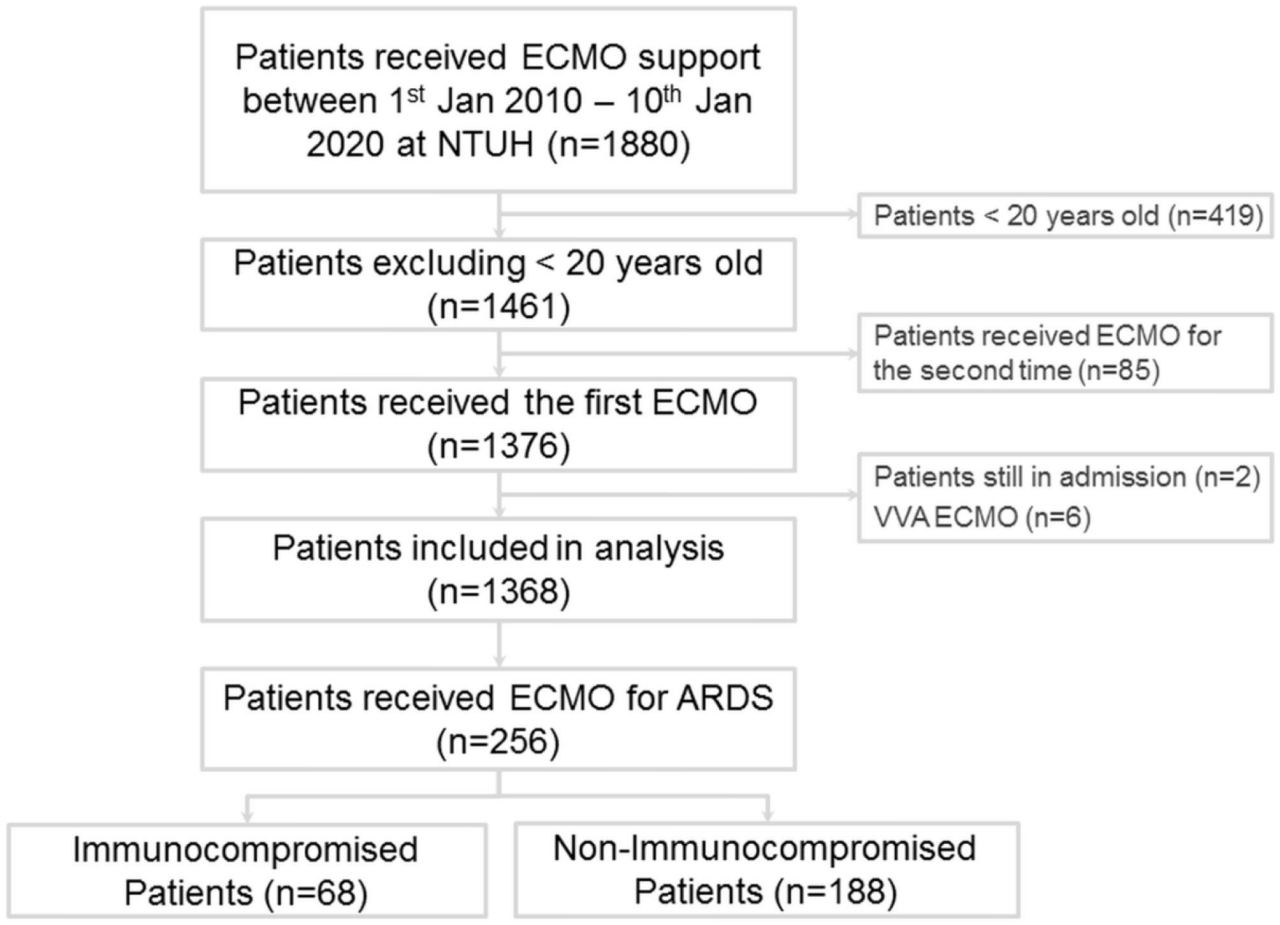
Flow diagram of patients with acute respiratory distress syndrome treated by extracorporeal membrane oxygenation.

During the study period, 1,880 patients received ECMO support at our institution (Figure 1), and 256 adults were enrolled in this study. Among these patients, 68 fulfilled the definition of immunocompromised status (Supplementary Table 1), including 13 with hematological malignancies (19.1%), 28 with active solid tumors (41.2%), 3 who received a solid organ transplant before receiving ECMO (4.4%), and 24 with autoimmune diseases (35.3%). The demographics and clinical characteristics of the patients are shown in Table 1. The immunocompromised patients had a lower baseline body mass index and higher modified Charlson comorbidity index than the immunocompetent patients. For the initial disease severity, both the inotropic score and APACHE II score were higher in the immunocompromised group than in the immunocompetent group. There was no significant difference in the modified APACHE II score between the two groups.

**Table 1:** Baseline characteristics of the patients grouped by immune status.

**Variables**	**Immunocompromised patients**	**Immunocompeten*t* patients**	***p* value**
	**(*N* = 68)**	**(*N* = 188)**	
Male sex, *n* (%)	44 (64.7)	137 (72.9)	0.216
Age, median (IQR)	59.8 (47.6–65.4)	56.7 (44.8–65.9)	0.377
Body mass index, median (IQR)	24.0 (21.5–27.5)	25.5 (23.1–29.2)	0.013^*^
VV ECMO, *n* (%)	63 (92.6)	160 (85.1)	0.140
Interval of MV to ECMO (hours), median (IQR)	54 (8.5–152.5)	34.5 (11–141.5)	0.702
Underlying diseases			
Charlson comorbidity index, median (IQR)	7 (4–10)	3 (2–5)	<0.001^*^
Modified Charlson comorbidity index, median (IQR)	5 (2–6)	3 (2–5)	0.019^*^
Congestive heart failure, *n* (%)	11 (16.2)	48 (25.5)	0.132
Hypertension, *n* (%)	30 (44.1)	79 (42.0)	0.776
Diabetes mellitus, *n* (%)	15 (22.1)	57 (30.3)	0.212
Coronary artery disease, *n* (%)	5 (7.3)	29 (15.4)	0.100
Remote stroke, *n* (%)	2 (2.9)	9 (4.8)	0.733
Cirrhosis of the liver, *n* (%)	4 (5.9)	8 (4.3)	0.525
Pre-ECMO dialysis, *n* (%)	2 (2.9)	2 (1.1)	0.288
Adjunctive treatment			
Neuromuscular blockers, *n* (%)	38 (55.9)	113 (60.1)	0.567
Prone position before ECMO, *n* (%)	9 (13.2)	19 (10.1)	0.499
iNO before ECMO, *n* (%)	22 (32.4)	45 (23.9)	0.199
Initial disease severity			
Severe ARDS, *n* (%)	59 (86.8)	168 (89.4)	0.655
APACHE II score, median (IQR)	23.5 (19.5–31)	20.0 (14–27)	0.006^*^
Modified APACHE II score, median (IQR)	21.5 (17.5–29.0)	20.0 (14–27)	0.198
Inotropic score, median (IQR)^**^	16.1 (0–36.0)	5 (0–28.5)	0.030)^*^
Ventilator setting			
Dynamic driving pressure (cmH2O), median (IQR)	17.0 (13–20)	18.0 (14–23)	0.088
Mechanical power (J/min), median (IQR)	22.7 (16.4–28.4)	24.8 (16.9–34.8)	0.246

* *p < 0.05*.

** *The inotropic score was calculated as 100 × epinephrine dose (μg/kg/min) + 100 × norepinephrine dose (μg/kg/min) + dopamine dose (μg/kg/min) + dobutamine dose (μg/kg/min)*. *APACHE, Acute Physiology and Chronic Health Evaluation; ARDS, acute respiratory distress syndrome; ECMO, extracorporeal membrane oxygenation; iNO, inhaled nitric oxide; IQR, interquartile range; MV, mechanical ventilation; SD, standard deviation; VV, venovenous*.

**Figure 2:**
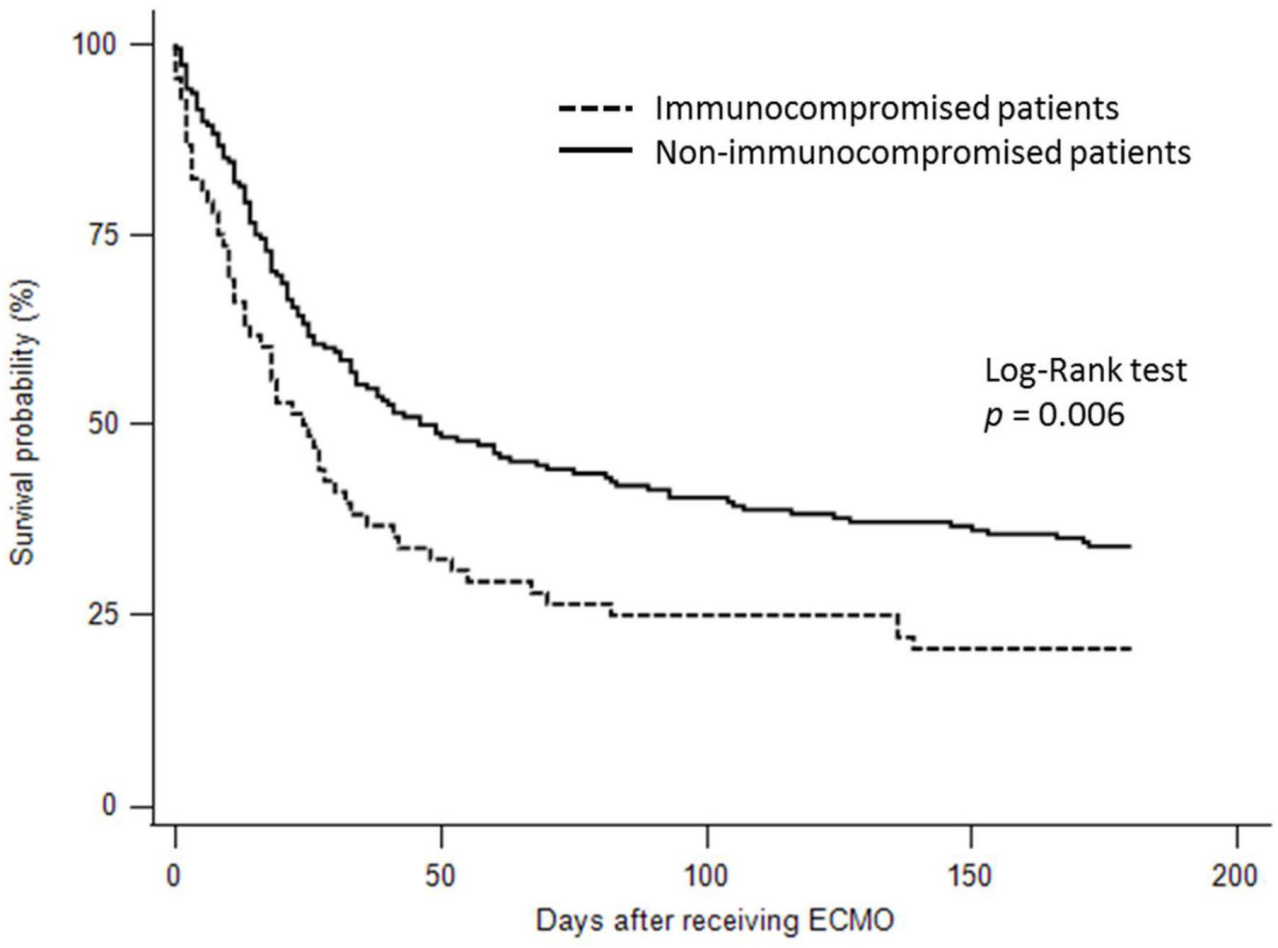
Kaplan-Meier survival curves for 6-month survival in the immunocompromised and immunocompetent patients with acute respiratory distress syndrome treated by extracorporeal membrane oxygenation.

For the clinical outcomes (Supplementary Table 2), crude comparisons showed that fewer immunocompromised patients were weaned from ECMO than immunocompetent patients (42.6 vs. 56.9%, *p* = 0.048). In addition, fewer immunocompromised patients survived to discharge than immunocompetent patients (19.1 vs. 42.6%, *p* < 0.001). The immunocompromised patients did not have significantly longer hospital length of stay than the immunocompetent patients (median 34 days with IQR 20–72.5 vs. median 29.5 days with IQR 18–57.5 days, *p* = 0.149). The ECMO duration in the immunocompromised patients were not significantly different from the immunocompetent patients (median 9.5 days with IQR 3.5–23.5 vs. median 13 days with IQR 6–21.5, *p* = 0.171). The 6-month survival rate in the immunocompromised patients was significantly lower than that in the immunocompetent patients (20.6 vs. 34.0%, log-rank test *p* = 0.006) (Figure 2).

We then compared the characteristics of the patients grouped by the status of ECMO weaning to identify possible risk factors associated with failure to wean from ECMO (Supplementary Table 3). The weaning failure group had a longer interval between intubation and receiving ECMO, more immunocompromised patients, higher modified Charlson comorbidity index, higher modified APACHE II score, more prone positioning before ECMO, more inhaled nitric oxide (iNO) use before ECMO, and higher inotropic score. Multivariable logistic regression analysis showed that a longer interval between intubation and receiving ECMO (odds ratio [OR] 1.09, 95% confidence interval [CI] 1.03–1.16), higher inotropic score (OR 1.02, 95% CI 1.01–1.03), and prone position before ECMO (OR 3.67, 95% CI 1.31–10.3) were independent risk factors of failure to wean from ECMO (Table 2). Immunocompromised status was not significantly associated with ECMO weaning failure (OR 1.41, 95% CI 0.76–2.63). To support our finding, we conduct propensity score analysis, matching the parameters related to the failure from weaning ECMO, including sex, body mass index, interval between intubation and ECMO cannulation, modified Charlson comorbidity index, modified APACHE II score, inotropic score, prone positioning before ECMO, and inhaled nitric oxide use before ECMO. After 1:1 propensity score matching, the logistic regression showed that the OR of immunocompromised status for weaning failure from ECMO was 0.99 (95% CI 0.50–2.02) (Supplementary Table 4). The characteristics of the cohort before and after the matching for each dataset created during multiple imputation was detailed in Supplementary Tables 5-1–5-10. This result was in line with the hypothesis that immunocompromised status was not an independent risk factor for failure to wean from ECMO.

**Table 2:** Risk factors of failure to wean from ECMO in the patients who received ECMO for ARDS by logistic regression.

**Risk factors**	**Univariable analysis**			**Multivariable analysis**		
	**OR**	**95% CI**	***p* value**	**OR**	**95% CI**	***p* value**
Male sex	0.60	0.35–1.02	0.061			
Body mass index	0.96	0.91–1.00	0.066			
Interval of MV to ECMO (per day)	1.11	1.06–1.17	<0.001^*^	1.09	1.03–1.16	0.002^*^
iNO before ECMO	1.86	1.06–3.27	0.032^*^	1.38	0.72–2.64	0.374^*^
Prone position before ECMO	4.86	1.90–12.5	<0.001^*^	3.67	1.31–10.3	0.014^*^
Mechanical power (J/min)	1.00	0.98–1.02	0.946			
Immunocompromised status	1.78	1.01–3.11	0.045^*^	1.41	0.76–2.63	0.279^*^
Modified Charlson comorbidity index	1.14	1.04–1.24	0.004^*^	1.07	0.97–1.18	0.186^*^
Modified APACHE II score	1.05	1.01–1.09	0.007^*^	1.00	0.96–1.04	0.977^*^
Inotropic score^**^	1.02	1.01–1.03	0.001^*^	1.02	1.01–1.03	0.002^*^

* *p < 0.05*.

** *The inotropic score was calculated as 100 × epinephrine dose (μg/kg/min) + 100 × norepinephrine dose (μg/kg/min) + dopamine dose (μg/kg/min) + dobutamine dose (μg/kg/min). APACHE, Acute Physiology and Chronic Health Evaluation; ARDS, acute respiratory distress syndrome; CI, confidence interval; ECMO, extracorporeal membrane oxygenation; iNO, inhaled nitric oxide; MV, mechanical ventilation; OR, odds ratio*.

To investigate why the prone position before ECMO use was associated with failure to wean from ECMO, we compared the characteristics of the patients grouped by whether or not they were in the prone position before ECMO. The prone position group had a longer interval between mechanical ventilation and receiving ECMO, higher rates of neuromuscular blocker and iNO use, and higher APACHE II score than the non-prone position group (Supplementary Table 5). Among the 28 patients receiving prone positioning, 21 patients underwent ECMO as rescue therapy within 2 days after prone positioning. The other seven patients improved with prone positioning, but they underwent ECMO directly when oxygenation deteriorated after discontinuing prone positioning. Complete data analysis showed similar results in the main analysis.

Among the immunocompromised patients, those with hematological malignancies had the numerically worst 6-month survival rate (7.7%) compared to the other immunocompromised patients (Supplementary Figure 1). With regards to weaning from ECMO, Fisher's exact test showed a significant difference between the different groups of immunocompromised patients (Table 3). The patients with autoimmune diseases (14/24, 58.3%) and those with a history of organ transplantation (3/3, 100%) had numerically higher weaning rates from ECMO than those with solid tumors or hematological malignancies. However, pairwise comparisons showed no significant differences after Bonferroni's correction. The duration of ECMO support and survival rate to discharge were not significantly different among the different groups of immunocompromised patients. Table 4 shows the clinical outcomes for specific causes of ARDS in the immunocompromised patients. The weaning rates from ECMO were numerically higher in those with pulmonary hemorrhage (6/8, 75%) and aspiration (5/9, 55.6%) than the other causes. Six of the patients with pulmonary hemorrhage were related to autoimmune diseases, and the other two patients had hematological malignancies. In contrast, the weaning rates were only 22.2% in the patients with interstitial lung disease (2/9) and 25% in the patients with sepsis (1/4). However, the survival rates were generally low in the immunocompromised patients (0–25.8%).

**Table 3:** Outcomes for specific immunocompromised status and specific causes of ARDS.

	**Hematological malignancy**	**Solid tumor**	**Organ transplantation**	**Autoimmune disease**	***p* value**
	**(*n* = 13)**	**(*n* = 28)**	**(*n* = 3)**	**(*n* = 24)**	
Weaned from ECMO, *n* (%)	2 (15.4)	10 (35.7)	3 (100)	14 (58.3)	0.009^*^
Duration of ECMO support, median (IQR)	10 (2–20.5)	10 (5–28)	9 (6.8–11.3)	8 (3–22)	0.897
Survival to discharge, *n* (%)	1 (7.7)	6 (21.4)	1 (33.3)	5 (20.8)	0.554

* *p < 0.05 in Fisher's exact test. Fisher's exact test showed a significant difference between the different groups of immunocompromised patients, but pairwise comparisons showed no significant differences after Bonferroni's correction*. *ARDS, acute respiratory distress syndrome; ECMO, extracorporeal membrane oxygenation; IQR, interquartile range*.

**Table 4:** Outcomes for specific causes of ARDS in the immunocompromised patients.

**Causes of ARDS**	**Total**	**Weaned from**	**Survival to**
		**ECMO**	**discharge**
Pneumonia, *n* (%)	31	13 (41.9)	8 (25.8)
Pulmonary hemorrhage, *n* (%)	8	6 (75)	1 (12.5)
Aspiration, *n* (%)	9	5 (55.6)	2 (22.2)
Interstitial lung disease, *n* (%)	9	2 (22.2)	1 (11.1)
Sepsis, *n* (%)	4	1 (25.0)	1 (25.0)
Others, *n* (%)	7	2 (28.6)	0 (0)

*ARDS, acute respiratory distress syndrome; ECMO, extracorporeal membrane oxygenation*.

## Discussion

In this study, the patients with ARDS who received ECMO and were immunocompromised had an overall worse general condition when initiating ECMO and subsequently worse clinical outcomes than those who were immunocompetent. However, after adjusting for potential confounders, immunocompromised status was not an independent risk factor for failure to wean from ECMO. In addition, the patients with autoimmune diseases and organ transplantation seemed to have a higher successful weaning rate from ECMO than those with hematological malignancies and solid cancer. With regards to the causes of ARDS, most of the patients who presented with pulmonary hemorrhage and aspiration could be weaned from ECMO. In contrast, most of the patients who presented with interstitial lung disease and sepsis died during ECMO.

We also found that in the patients who received ECMO for ARDS, those who were immunocompromised had a higher inotropic score, higher APACHE II score, and higher Charlson comorbidity index than those who were immunocompetent. These findings are consistent with a study by Na et al. who reported that the general condition of immunocompromised patients was poorer than immunocompetent patients (5). We further showed that immunocompromised status was not independently associated with failure to wean from ECMO in multivariable analysis. This finding could support the decision to use ECMO in immunocompromised patients with ARDS. In a report by Wohlfarth et al. among patients with hematological malignancy and acute respiratory failure, ECMO was successfully weaned in 10 out of 17 episodes (59%), and seven patients survived until discharge (24). A series of case reports have also suggested the potential role of ECMO as a bridge to curative chemotherapy in patients with hematological malignancies (25). For patients with autoimmune diseases, several studies have also reported that ECMO may serve as a bridge to immunomodulation in patients with pulmonary hemorrhage related to ANCA-associated vasculitis and systemic lupus erythematous (26, 27). Because patients with immunosuppressive diseases have worse long-term outcomes than the general population, the weaning rate from ECMO may be a more practical index to evaluate the benefits of ECMO in these patients than other traditional clinical indexes.

Among the immunocompromised patients in the present study, those with autoimmune diseases seemed to have a higher weaning rate from ECMO than those with solid tumors and hematological malignancies. In addition, the weaning rates from ECMO seemed to be different for the different causes of ARDS. For patients with interstitial lung diseases, the weaning rate from ECMO was only 22.2%, which is compatible with a report by Trudzinski et al. (28). In their retrospective analysis, 21 patients with interstitial lung diseases and acute respiratory failure received ECMO support, and 15 patients did not receive a lung transplantation during ECMO support. Among these 15 patients, 14 died during ECMO. ECMO was withdrawn in five patients, and the others died because of sepsis-related multi-organ failure. Therefore, if it is not a bridge to lung transplantation, ECMO may not be a reasonable rescue therapy for patients with interstitial lung disease-related ARDS.

On the other hand, pulmonary hemorrhage related to autoimmune diseases such as systemic lupus erythematosus or ANCA-associated vasculitis has been shown to respond to glucocorticoids and immunosuppressive agents (29). Therefore, for patients with autoimmune diseases complicated with pulmonary hemorrhage and ARDS, ECMO may be indicated as a bridge to the effects of glucocorticoids and immunosuppressive agents (3). A series of case studies have reported successful rescue by ECMO in patients with autoimmune diseases complicated with refractory pulmonary hemorrhage (27). However, we also found that although most patients with autoimmune disease-related pulmonary hemorrhage could be weaned from ECMO, the rate of survival to discharge was low. Most of these patients died in hospital due to severe and recurrent infections. Therefore, how to avoid overwhelming life-threatening infections while prescribing adequate immunosuppressive agents is an essential issue in these patients. More data is needed not only from successful cases but also from patients who die during admission to delineate the practical treatment strategy for these patients. In addition, our results showed that the weaning rates from ECMO and survival rates to discharge in the patients with autoimmune diseases with pneumonia were promising. Although these results should be interpreted with caution due to the small number of cases, our findings may support the use of ECMO in patients with autoimmune diseases who present with ARDS related to pneumonia.

Unexpectedly, we found that undergoing prone positioning before ECMO use was independently associated with failure to wean from ECMO in this study. We suggest interpreting this result cautiously. Many studies suggested that prone positioning decreases mortality in severe ARDS patients (30, 31). In patients receiving ECMO for ARDS, prone positioning before ECMO use may also provide a protective effect (13, 32). Medical societies also suggested using prone positioning to treat patients with severe ARDS (33). However, in the real-world, prone positioning was not used widely. Less than 20% of severe ARDS patients received prone positioning in an international, multi-center, prospective cohort study (34). In this study, only 10.9% of patients received prone positioning before ECMO, and the small number of cases limited the interpretation of this result. Second, because this study was retrospective, we did not know what prompted physicians to use prone positioning before ECMO or choose ECMO directly in ARDS patients. Furthermore, among patients treated by prone position before ECMO, up to 75% (21 out of 28 patients) patients had interval between prone positioning and ECMO cannulation <2 days. These patients underwent ECMO as a rescue therapy from failure in prone position, and therefore might have poorer prognosis than other patients. Therefore, we suggest interpreting this finding conservatively. For clarifying the impact of prone positioning before ECMO use in ARDS patients, incorporating prone positioning as one of the inclusion criteria for the comparison group in the future study may help answer the issue (35).

This study also found that increasing duration of ventilation before ECMO and increasing level of the inotropic score were independently associated with failure to wean from ECMO. In line with our finding, previous studies reported that longer ventilation duration before ECMO was independently associated with higher in-hospital mortality for patients receiving ECMO for acute respiratory failure or ARDS (14, 36, 37). Besides, Schmidt et al. also found that longer ventilation duration before ECMO was an independent risk factor for 6-month mortality (13). Therefore, according to these findings, early ECMO implementation in patients with severe ARDS may improve the prognosis. Although the EOLIA trial did not found a significant difference in survival rates between using ECMO as a frontline treatment or as a rescue treatment, the limitation in its design warrants further studies to define the time window for implementing ECMO in these patients (1). For the association between inotropic agents and the prognosis, Brogan et al. found no significant difference in rates of inotropic agents before receiving ECMO for respiratory failure between survivors and non-survivors during admission (37). Schmidt et al. also reported no apparent association between inotropic agent use and survival status at 6 months (13). In contrast, our study showed that the increasing level of the inotropic score was independently associated with failure to wean from ECMO. The difference between this study and previous literature might be due to different measures of inotropic agents. In this study, we used the inotropic score to quantify cardiovascular support by inotropic agents (23, 38). In comparison with reporting only whether using inotropic agents, the inotropic score provides an estimation of the amount of cardiovascular support and is regarded as a surrogate marker of illness severity and an intermediate predictor of eventual clinical outcome (39).

Our study has several limitations. First, the numbers of patients with specific immunocompromised statuses and specific causes of ARDS were small. However, the trends of clinical outcomes in the patients with specific causes of ARDS, such as pulmonary hemorrhage and interstitial lung disease are consistent with previous studies. This consistency suggests the plausibility of our results, and could suggest further directions for clinical practice and research. Second, although patients with ARDS at our institute are treated using a lung protection strategy, we did not report daily detailed mechanical ventilation settings during ECMO. However, Schmidt el. reported that even though an ultraprotective lung ventilation strategy while on ECMO was widely used, the mechanical ventilation settings during the first two days of ECMO support did not impact the patients' prognosis (40). Third, because of the retrospective design, we could not systematically evaluate how severely the patients were immunocompromised. Until recently, there has been no consensus on stratifying the competency of the immune system. Therefore researchers could only use disease categories such as hematological cancer, active solid tumors, and autoimmune diseases to classify the immune status. This classification oversimplifies the immunological status, and therefore causes heterogeneity in the analysis.

In conclusion, our study suggests that immunocompromised status was not an independent risk factor of failure to wean from ECMO in patients with ARDS. For patients with pulmonary hemorrhage and aspiration-related ARDS, ECMO might be considered as a bridge therapy for therapeutic purposes.

## Data Availability Statement

Data are available upon reasonable request.

## Ethics Statement

The studies involving human participants were reviewed and approved by National Taiwan University Hospital. Written informed consent for participation was not required for this study in accordance with the national legislation and the institutional requirements.

## Author Contributions

C-FC, Y-YC, and C-HW: patient care, study design, data analysis, and writing. M-CS: study design, statistics, data analysis, and writing. Y-MH, T-YL, C-HLu, S-CH, K-JL, N-HC, H-YY, and Y-SC: patient care and data analysis. L-JT and C-HLa: patient care, database maintenance, and data analysis. All authors provided a substantial contribution to the conception, design and interpretation of the work, drafted the work or revised it critically for important intellectual content, and provided final approval of the submitted version of the manuscript.

## Funding

This work was supported by the Ministry of Science and Technology of Taiwan [grant numbers 106-2314-B-002-155-MY3, 109-2314-B-002-218, and 110-2314-B-002-230], National Taiwan University Hospital [grant number NTUH-109-S4586], and National Taiwan University Hospital Yun-Lin branch [grant number NTUHYL110.N001].

## Conflict of Interest

The authors declare that the research was conducted in the absence of any commercial or financial relationships that could be construed as a potential conflict of interest.

## Publisher's Note

All claims expressed in this article are solely those of the authors and do not necessarily represent those of their affiliated organizations, or those of the publisher, the editors and the reviewers. Any product that may be evaluated in this article, or claim that may be made by its manufacturer, is not guaranteed or endorsed by the publisher.
