# Supplementary material for: Extracorporeal Membrane Oxygenation in Immunocompromised Patients With Acute Respiratory Distress Syndrome—A Retrospective Cohort Study
Source: Front Med (Lausanne). 2021 Dec 3;8:755147. doi: 10.3389/fmed.2021.755147 (PMC8679966; doi:10.3389/fmed.2021.755147)
Supplement: Supplementary file 1 [file Data_Sheet_1.docx]

Supplementary Material

# Supplementary Table 1. Categories of the immunocompromised patients

# Supplementary Table 2. Clinical outcomes of the patients who received ECMO due to ARDS

# Supplementary Table 3. Baseline characteristics of the patients grouped by the status of weaning from ECMO

**Supplementary Table 4** The odds ratio of immunocompromised status for failure of weaning from ECMO in each dataset from the multiple imputation and propensity score analysis.

# Supplementary Table 5-1 ~ 5-10. Baseline characteristic of patients in datasets created by multiple imputation before and after propensity score matching

# Supplementary Table 6. Baseline characteristics of the patients grouped by the use of the prone position before ECMO

# Supplementary Figure 1. Kaplan-Meier survival curves for 6-month survival in the immunocompromised patients with different causes of immunocompromised status

| **Supplementary Table 1** Categories of the immunocompromised patients | |
| --- | --- |
| Immunocompromised status | Immunocompromised patients (N = 68) |
| Hematological malignancy, n (%) | 13 (19.1) |
| Leukemia | 7 |
| Lymphoma | 5 |
| Others | 1 |
| Solid tumor, n (%) | 28 (41.2) |
| Lung cancer | 11 |
| Esophageal cancer | 8 |
| Colon cancer | 2 |
| Others | 7 |
| Solid organ transplant, n (%) | 3 (4.4) |
| Kidney | 2 |
| Heart | 1 |
| Autoimmune diseases, n (%) | 24 (35.3) |
| Systemic lupus erythematosus | 6 |
| Rheumatoid arthritis | 5 |
| ANCA-associated vasculitis | 4 |
| Sjögren’s syndrome | 4 |
| Ankylosing spondylitis | 3 |
| Others | 2 |

| **Supplementary Table 2** Clinical outcomes of the patients who received ECMO due to ARDS | | | |
| --- | --- | --- | --- |
| Clinical outcomes | Immunocompromised patients (N = 68) | Immunocompetent patients (N = 188) | *p* value |
| Weaned from ECMO, n (%) | 29 (42.6) | 107 (56.9) | 0.048* |
| Survival to discharge, n (%) | 13 (19.1) | 80 (42.6) | <0.001* |
| Hospital length of stay (days), median (IQR) | 34 (20 – 72.5) | 29.5 (18 – 57.5) | 0.149 |
| ECMO duration, (days), median (IQR) | 9.5 (3.5 – 23.5) | 13 (6 – 21.5) | 0.171 |
| **p* < 0.05  ARDS, acute respiratory distress syndrome; ECMO, extracorporeal membrane oxygenation; IQR, interquartile range | | | |

| **Supplementary Table 3** Baseline characteristics of the patients grouped by the status of weaning from ECMO | | | |
| --- | --- | --- | --- |
| Variables | ECMO weaning success  (N = 136) | ECMO weaning failure  (N = 120) | *p* value |
| Male sex, n (%) | 103 (75.7) | 78 (65) | 0.074 |
| Age, median (IQR) | 55.4 (43.2 – 65.5) | 59.8 (48 – 66.1) | 0.157 |
| Body mass index, median (IQR) | 25.4 (23.4 – 29.1) | 24.8 (22.0 – 28.0) | 0.090 |
| VV ECMO, n (%) | 118 (86.8) | 105 (87.5) | 1.000 |
| Interval of MV to ECMO (hours), median (IQR) | 24 (7.5 – 75.5) | 77 (23 – 226.5) | < 0.001* |
| Immunocompromised, n (%) | 29 (21.3) | 39 (32.5) | 0.048* |
| Underlying diseases |  |  |  |
| Charlson comorbidity index, median (IQR) | 3 (2 – 6) | 5 (3 – 8) | < 0.001* |
| Modified Charlson comorbidity index, median (IQR) | 3 (1.5 – 5) | 4 (2 – 6) | 0.005* |
| Congestive heart failure, n (%) | 34 (25) | 25 (20.8) | 0.460 |
| Hypertension, n (%) | 61 (44.9) | 48 (40) | 0.450 |
| Diabetes mellitus, n (%) | 38 (27.9) | 34 (28.3) | 1.000 |
| Coronary artery disease, n (%) | 19 (14.0) | 15 (12.5) | 0.854 |
| Remote stroke, n (%) | 5 (3.7) | 6 (5) | 0.760 |
| Cirrhosis of the liver, n (%) | 5 (3.7) | 7 (5.8) | 0.556 |
| Pre-ECMO dialysis, n (%) | 3 (2.2) | 1 (0.8) | 0.625 |
| Adjunctive treatment |  |  |  |
| Neuromuscular blockers, n (%) | 78 (57.4) | 73 (60.8) | 0.612 |
| Prone position before ECMO, n (%) | 6 (4.4) | 22 (18.3) | < 0.001* |
| iNO before ECMO, n (%) | 28 (20.6) | 39 (32.5) | 0.033* |
| Initial disease severity |  |  |  |
| Severe ARDS, n (%) | 117 (86.0) | 110 (91.7) | 0.172 |
| APACHE II score, median (IQR) | 19 (14 – 25) | 25 (20 – 29) | < 0.001* |
| Modified APACHE II score, median (IQR) | 18.5 (14 – 25) | 23.5 (18 – 29) | 0.002* |
| Inotropic score, median (IQR)** | 3 (0 – 16.8) | 16 (0 – 39.9) | < 0.001* |
| Ventilator setting |  |  |  |
| Dynamic driving pressure (cmH2O), median (IQR) | 18 (14 – 21) | 18 (14 – 23) | 0.563 |
| Mechanical power (J/min), median (IQR) | 22.9 (16.9 – 32.9) | 25.5 (15.7 – 34.3) | 0.598 |
| **p* < 0.05  **The inotropic score was calculated as 100 × epinephrine dose (μg/kg/min) + 100 × norepinephrine dose (μg/kg/min) + dopamine dose (μg/kg/min) + dobutamine dose (μg/kg/min)  APACHE, Acute Physiology and Chronic Health Evaluation; ARDS, acute respiratory distress syndrome; ECMO, extracorporeal membrane oxygenation; iNO, inhaled nitric oxide; IQR, interquartile range; MV, mechanical ventilation; SD, standard deviation; VV, venovenous | | | |

| **Supplementary Table 4** The odds ratio of immunocompromised status for failure of weaning from ECMO in each dataset from the multiple imputation and propensity score analysis. | | | |
| --- | --- | --- | --- |
| Datasets created by multiple imputation | β | Standard error | Odds ratio |
| 1 | 0.152 | 0.389 | 1.164 |
| 2 | 0.163 | 0.404 | 1.178 |
| 3 | 0.405 | 0.404 | 1.5 |
| 4 | 0.149 | 0.386 | 1.161 |
| 5 | -0.305 | 0.391 | 0.737 |
| 6 | -0.559 | 0.402 | 0.572 |
| 7 | -0.237 | 0.398 | 0.789 |
| 8 | 0.000 | 0.395 | 1.000 |
| 9 | -0.245 | 0.405 | 0.783 |
| 10 | 0.373 | 0.433 | 1.453 |
| Pooled | -0.010 | 0.355 | 0.990 |

| **Supplementary Table 5-1** Baseline characteristic of patients in dataset 1 created by multiple imputation before and after propensity score matching (PSM) | | | | | | |
| --- | --- | --- | --- | --- | --- | --- |
| Variables | Before PSM | | | After PSM | | |
|  | Immunocompromised patients (N = 68) | Immunocompetent patients (N = 188) | Standardized difference, % | Immunocompromised patients (N = 54) | Immunocompetent patients (N = 54) | Standardized difference, % |
| Male sex, % | 64.70% | 72.90% | -18% | 66.7% | 63% | 8% |
| Age, mean | 56.7 | 55.1 | 1% | 57.4 | 57.5 | 0% |
| Body mass index, mean | 24.7 | 26.4 | -6% | 25.1 | 25.5 | -3% |
| VV ECMO, % | 92.60% | 85.10% | 24% | 92.60% | 87% | 19% |
| Interval of MV to ECMO (hours), mean | 128.8 | 103.5 | 0% | 123.9 | 96.1 | 0% |
| Modified Charlson comorbidity index, mean | 4.7 | 3.9 | 9% | 4.3 | 4.5 | -2% |
| Prone position before ECMO, % | 13.20% | 10.10% | 10% | 13% | 13% | 0% |
| iNO before ECMO, % | 32.40% | 23.90% | 19% | 29.6% | 33.3% | -8% |
| Modified APACHE II score, mean | 22.3 | 21 | 2% | 21.5 | 21.3 | 0% |
| Inotropic score, mean | 23.1 | 19.3 | 0% | 22.5 | 18.5 | 0% |
| Dynamic driving pressure (cmH2O) , mean | 16.4 | 18.8 | -5% | 16.2 | 17.9 | -4% |
| Mechanical power (J/min) , mean | 26.7 | 29.5 | -1% | 27.2 | 29.5 | -1% |
| **p* < 0.05  APACHE, Acute Physiology and Chronic Health Evaluation; ARDS, acute respiratory distress syndrome; ECMO, extracorporeal membrane oxygenation; iNO, inhaled nitric oxide; IQR, interquartile range; MV, mechanical ventilation; SD, standard deviation; VV, venovenous | | | | | | |
| **Supplementary Table 5-2** Baseline characteristic of patients in dataset 2 created by multiple imputation before and after propensity score matching (PSM) | | | | | | |
| Variables | Before PSM | | | After PSM | | |
|  | Immunocompromised patients (N = 68) | Immunocompetent patients (N = 188) | Standardized difference, % | Immunocompromised patients (N = 49) | Immunocompetent patients (N = 49) | Standardized difference, % |
| Male sex, % | 64.70% | 72.90% | -18% | 67.3% | 71.4% | -9% |
| Age, mean | 56.7 | 55.1 | 1% | 57.6 | 52.8 | 2% |
| Body mass index, mean | 24.3 | 26.3 | -7% | 25.1 | 25.1 | 0% |
| VV ECMO, % | 92.60% | 85.10% | 24% | 91.8% | 83.7% | 25% |
| Interval of MV to ECMO (hours), mean | 128.8 | 103.5 | 0% | 122.3 | 118 | 0% |
| Modified Charlson comorbidity index, mean | 4.7 | 3.9 | 9% | 4.4 | 4.1 | 4% |
| Prone position before ECMO, % | 13.20% | 10.10% | 10% | 14.3% | 12.2% | 6% |
| iNO before ECMO, % | 32.40% | 23.90% | 19% | 24.5% | 32.7% | -18% |
| Modified APACHE II score, mean | 22.4 | 21.2 | 2% | 22.1 | 22.8 | -1% |
| Inotropic score, mean | 24.5 | 19.5 | 1% | 19.3 | 20.1 | 0% |
| Dynamic driving pressure (cmH2O) , mean | 17.5 | 18.6 | -2% | 17.6 | 19.6 | -3% |
| Mechanical power (J/min) , mean | 27.7 | 29.5 | -1% | 28.2 | 32.3 | -1% |
| **p* < 0.05  APACHE, Acute Physiology and Chronic Health Evaluation; ARDS, acute respiratory distress syndrome; ECMO, extracorporeal membrane oxygenation; iNO, inhaled nitric oxide; IQR, interquartile range; MV, mechanical ventilation; SD, standard deviation; VV, venovenous | | | | | | |
| **Supplementary Table 5-3** Baseline characteristic of patients in dataset 3 created by multiple imputation before and after propensity score matching (PSM) | | | | | | |
| Variables | Before PSM | | | After PSM | | |
|  | Immunocompromised patients (N = 68) | Immunocompetent patients (N = 188) | Standardized difference, % | Immunocompromised patients (N = 50) | Immunocompetent patients (N = 50) | Standardized difference, % |
| Male sex, % | 64.70% | 72.90% | -18% | 72% | 60% | 26% |
| Age, mean | 56.7 | 55.1 | 1% | 57.5 | 53.6 | 2% |
| Body mass index, mean | 24.6 | 26.4 | -7% | 25.2 | 24.9 | 2% |
| VV ECMO, % | 92.60% | 85.10% | 24% | 90% | 82% | 23% |
| Interval of MV to ECMO (hours), mean | 128.8 | 103.5 | 0% | 138.9 | 129.2 | 0% |
| Modified Charlson comorbidity index, mean | 4.7 | 3.9 | 9% | 4.3 | 3.4 | 13% |
| Prone position before ECMO, % | 13.20% | 10.10% | 10% | 14% | 10% | 12% |
| iNO before ECMO, % | 32.40% | 23.90% | 19% | 32% | 26% | 13% |
| Modified APACHE II score, mean | 22.2 | 20.9 | 2% | 21 | 21.1 | 0% |
| Inotropic score, mean | 23.4 | 20.3 | 0% | 19.8 | 30.8 | -1% |
| Dynamic driving pressure (cmH2O) , mean | 16.6 | 18.6 | -4% | 16.8 | 17.9 | -3% |
| Mechanical power (J/min) , mean | 23.9 | 28.7 | -2% | 22.1 | 30.9 | -2% |
| **p* < 0.05  APACHE, Acute Physiology and Chronic Health Evaluation; ARDS, acute respiratory distress syndrome; ECMO, extracorporeal membrane oxygenation; iNO, inhaled nitric oxide; IQR, interquartile range; MV, mechanical ventilation; SD, standard deviation; VV, venovenous | | | | | | |
| **Supplementary Table 5-4** Baseline characteristic of patients in dataset 4 created by multiple imputation before and after propensity score matching (PSM) | | | | | | |
| Variables | Before PSM | | | After PSM | | |
|  | Immunocompromised patients (N = 68) | Immunocompetent patients (N = 188) | Standardized difference, % | Immunocompromised patients (N = 54) | Immunocompetent patients (N = 54) | Standardized difference, % |
| Male sex, % | 64.70% | 72.90% | -18% | 70.4% | 74.1% | -8% |
| Age, mean | 56.7 | 55.1 | 1% | 57.3 | 56.9 | 0% |
| Body mass index, mean | 24.6 | 26.4 | -7% | 24.8 | 24.8 | 0% |
| VV ECMO, % | 92.60% | 85.10% | 24% | 92.6% | 81.5% | 34% |
| Interval of MV to ECMO (hours), mean | 128.8 | 103.5 | 0% | 137.8 | 131.0 | 0% |
| Modified Charlson comorbidity index, mean | 4.7 | 3.9 | 9% | 4.4 | 4.5 | -1% |
| Prone position before ECMO, % | 13.20% | 10.10% | 10% | 13.0% | 14.8% | -5% |
| iNO before ECMO, % | 32.40% | 23.90% | 19% | 27.8% | 33.3% | -12% |
| Modified APACHE II score, mean | 22.3 | 21.1 | 2% | 22 | 22.5 | -1% |
| Inotropic score, mean | 23.6 | 19 | 0% | 22.1 | 17.1 | 1% |
| Dynamic driving pressure (cmH2O) , mean | 16.4 | 18.3 | -4% | 15.8 | 19.0 | -4% |
| Mechanical power (J/min) , mean | 25.4 | 29 | -1% | 25.1 | 34.0 | -3% |
| **p* < 0.05  APACHE, Acute Physiology and Chronic Health Evaluation; ARDS, acute respiratory distress syndrome; ECMO, extracorporeal membrane oxygenation; iNO, inhaled nitric oxide; IQR, interquartile range; MV, mechanical ventilation; SD, standard deviation; VV, venovenous | | | | | | |
| **Supplementary Table 5-5** Baseline characteristic of patients in dataset 5 created by multiple imputation before and after propensity score matching (PSM) | | | | | | |
| Variables | Before PSM | | | After PSM | | |
|  | Immunocompromised patients (N = 68) | Immunocompetent patients (N = 188) | Standardized difference, % | Immunocompromised patients (N = 53) | Immunocompetent patients (N = 53) | Standardized difference, % |
| Male sex, % | 64.70% | 72.90% | -18% | 67.9% | 66^ | 4% |
| Age, mean | 56.7 | 55.1 | 1% | 58.6 | 55.1 | 2% |
| Body mass index, mean | 24.3 | 26.4 | -8% | 24.8 | 25.3 | -3% |
| VV ECMO, % | 92.60% | 85.10% | 24% | 92.5% | 77.4% | 43% |
| Interval of MV to ECMO (hours), mean | 128.8 | 103.5 | 0% | 122.3 | 105.1 | 0% |
| Modified Charlson comorbidity index, mean | 4.7 | 3.9 | 9% | 4.5 | 4.5 | 0% |
| Prone position before ECMO, % | 13.20% | 10.10% | 10% | 13.2% | 5.7% | 26% |
| iNO before ECMO, % | 32.40% | 23.90% | 19% | 26.4% | 32.1% | -13% |
| Modified APACHE II score, mean | 22.2 | 21.1 | 2% | 22.7 | 20.9 | 3% |
| Inotropic score, mean | 23.7 | 19.4 | 0% | 23.2 | 27.2 | 0% |
| Dynamic driving pressure (cmH2O) , mean | 16.4 | 18.6 | -4% | 16.0 | 19.7 | -9% |
| Mechanical power (J/min) , mean | 24.6 | 28.6 | -2% | 24.0 | 29.4 | -2% |
| **p* < 0.05  APACHE, Acute Physiology and Chronic Health Evaluation; ARDS, acute respiratory distress syndrome; ECMO, extracorporeal membrane oxygenation; iNO, inhaled nitric oxide; IQR, interquartile range; MV, mechanical ventilation; SD, standard deviation; VV, venovenous | | | | | | |
| **Supplementary Table 5-6** Baseline characteristic of patients in dataset 6 created by multiple imputation before and after propensity score matching (PSM) | | | | | | |
| Variables | Before PSM | | | After PSM | | |
|  | Immunocompromised patients (N = 68) | Immunocompetent patients (N = 188) | Standardized difference, % | Immunocompromised patients (N = 52) | Immunocompetent patients (N = 52) | Standardized difference, % |
| Male sex, % | 64.70% | 72.90% | -18% | 69.2% | 73.1% | -9% |
| Age, mean | 56.7 | 55.1 | 1% | 56.7 | 56.2 | 0% |
| Body mass index, mean | 24.6 | 26.4 | -7% | 25.2 | 25.3 | -1% |
| VV ECMO, % | 92.60% | 85.10% | 24% | 90.4% | 80.8% | 28% |
| Interval of MV to ECMO (hours), mean | 128.8 | 103.5 | 0% | 121.1 | 115.6 | 0% |
| Modified Charlson comorbidity index, mean | 4.7 | 3.9 | 9% | 4.6 | 4.4 | 2% |
| Prone position before ECMO, % | 13.20% | 10.10% | 10% | 13.5% | 13.5% | 0% |
| iNO before ECMO, % | 32.40% | 23.90% | 19% | 28.8% | 36.5% | -16% |
| Modified APACHE II score, mean | 22.4 | 20.8 | 3% | 22.3 | 22.1 | 0% |
| Inotropic score, mean | 23.3 | 19.8 | 0% | 21.6 | 25.5 | 0% |
| Dynamic driving pressure (cmH2O) , mean | 16.5 | 18.5 | -4% | 16.7 | 19.3 | -3% |
| Mechanical power (J/min) , mean | 26.0 | 28.8 | -1% | 25.7 | 31.9 | -2% |
| **p* < 0.05  APACHE, Acute Physiology and Chronic Health Evaluation; ARDS, acute respiratory distress syndrome; ECMO, extracorporeal membrane oxygenation; iNO, inhaled nitric oxide; IQR, interquartile range; MV, mechanical ventilation; SD, standard deviation; VV, venovenous | | | | | | |
| **Supplementary Table 5-7** Baseline characteristic of patients in dataset 7 created by multiple imputation before and after propensity score matching (PSM) | | | | | | |
| Variables | Before PSM | | | After PSM | | |
|  | Immunocompromised patients (N = 68) | Immunocompetent patients (N = 188) | Standardized difference, % | Immunocompromised patients (N = 51) | Immunocompetent patients (N = 51) | Standardized difference, % |
| Male sex, % | 64.70% | 72.90% | -18% | 66.7% | 64.7% | 4% |
| Age, mean | 56.7 | 55.1 | 1% | 56.2 | 51.8 | 2% |
| Body mass index, mean | 24.6 | 26.4 | -7% | 25.1 | 24.7 | 2% |
| VV ECMO, % | 92.60% | 85.10% | 24% | 90.2% | 82.4% | 23% |
| Interval of MV to ECMO (hours), mean | 128.8 | 103.5 | 0% | 117.9 | 105.6 | 0% |
| Modified Charlson comorbidity index, mean | 4.7 | 3.9 | 9% | 21.4 | 22.5 | -2% |
| Prone position before ECMO, % | 13.20% | 10.10% | 10% | 15.7% | 3.9% | 40% |
| iNO before ECMO, % | 32.40% | 23.90% | 19% | 29.4% | 21.6% | 18% |
| Modified APACHE II score, mean | 22.2 | 21.0 | 2% | 21.4 | 22.5 | -2% |
| Inotropic score, mean | 23.2 | 20.2 | 0% | 21.9 | 26.6 | 0% |
| Dynamic driving pressure (cmH2O) , mean | 16.8 | 18.3 | -3% | 16.2 | 18 | -5% |
| Mechanical power (J/min) , mean | 24.0 | 28.2 | -2% | 23.2 | 29.6 | -2% |
| **p* < 0.05  APACHE, Acute Physiology and Chronic Health Evaluation; ARDS, acute respiratory distress syndrome; ECMO, extracorporeal membrane oxygenation; iNO, inhaled nitric oxide; IQR, interquartile range; MV, mechanical ventilation; SD, standard deviation; VV, venovenous | | | | | | |
| **Supplementary Table 5-8** Baseline characteristic of patients in dataset 8 created by multiple imputation before and after propensity score matching (PSM) | | | | | | |
| Variables | Before PSM | | | After PSM | | |
|  | Immunocompromised patients (N = 68) | Immunocompetent patients (N = 188) | Standardized difference, % | Immunocompromised patients (N = 52) | Immunocompetent patients (N = 52) | Standardized difference, % |
| Male sex, % | 64.70% | 72.90% | -18% | 71.2% | 71.2% | 0% |
| Age, mean | 56.7 | 55.1 | 1% | 59.2 | 56.2 | 1% |
| Body mass index, mean | 24.6 | 26.4 | -7% | 25 | 25.1 | -1% |
| VV ECMO, % | 92.60% | 85.10% | 24% | 94.2% | 86.5% | 26% |
| Interval of MV to ECMO (hours), mean | 128.8 | 103.5 | 0% | 140.4 | 122.8 | 0% |
| Modified Charlson comorbidity index, mean | 4.7 | 3.9 | 9% | 4.7 | 4 | 9% |
| Prone position before ECMO, % | 13.20% | 10.10% | 10% | 11.5% | 13.5% | -6% |
| iNO before ECMO, % | 32.40% | 23.90% | 19% | 26.9% | 36.5% | -21% |
| Modified APACHE II score, mean | 22.1 | 21 | 2% | 22.0 | 21.3 | 1% |
| Inotropic score, mean | 23.9 | 19.8 | 0% | 22.6 | 25.9 | 0% |
| Dynamic driving pressure (cmH2O) , mean | 16.4 | 18.7 | -5% | 15.7 | 20.1 | -6% |
| Mechanical power (J/min) , mean | 27.1 | 29.7 | -1% | 27.2 | 34.2 | -2% |
| **p* < 0.05  APACHE, Acute Physiology and Chronic Health Evaluation; ARDS, acute respiratory distress syndrome; ECMO, extracorporeal membrane oxygenation; iNO, inhaled nitric oxide; IQR, interquartile range; MV, mechanical ventilation; SD, standard deviation; VV, venovenous | | | | | | |
| **Supplementary Table 5-9** Baseline characteristic of patients in dataset 9 created by multiple imputation before and after propensity score matching (PSM) | | | | | | |
| Variables | Before PSM | | | After PSM | | |
|  | Immunocompromised patients (N = 68) | Immunocompetent patients (N = 188) | Standardized difference, % | Immunocompromised patients (N = 50) | Immunocompetent patients (N = 50) | Standardized difference, % |
| Male sex, % | 64.70% | 72.90% | -18% | 70% | 66% | 9% |
| Age, mean | 56.7 | 55.1 | 1% | 58.0 | 54.9 | 1% |
| Body mass index, mean | 24.5 | 26.4 | -7% | 24.5 | 24.7 | -1% |
| VV ECMO, % | 92.60% | 85.10% | 24% | 94.0% | 88.0% | 21% |
| Interval of MV to ECMO (hours), mean | 128.8 | 103.5 | 0% | 128.7 | 128.2 | 0% |
| Modified Charlson comorbidity index, mean | 4.7 | 3.9 | 9% | 4.8 | 4.4 | 4% |
| Prone position before ECMO, % | 13.20% | 10.10% | 10% | 12% | 12% | 0% |
| iNO before ECMO, % | 32.40% | 23.90% | 19% | 30% | 32% | -4% |
| Modified APACHE II score, mean | 22.4 | 20.9 | 3% | 21.9 | 22.2 | -1% |
| Inotropic score, mean | 23.4 | 19.9 | 0% | 23.2 | 30.8 | 0% |
| Dynamic driving pressure (cmH2O) , mean | 17.1 | 18.3 | -2% | 17.2 | 18.4 | -1% |
| Mechanical power (J/min) , mean | 24.9 | 28.7 | -2% | 24.0 | 27.4 | -1% |
| **p* < 0.05  APACHE, Acute Physiology and Chronic Health Evaluation; ARDS, acute respiratory distress syndrome; ECMO, extracorporeal membrane oxygenation; iNO, inhaled nitric oxide; IQR, interquartile range; MV, mechanical ventilation; SD, standard deviation; VV, venovenous | | | | | | |
| **Supplementary Table 5-10** Baseline characteristic of patients in dataset 10 created by multiple imputation before and after propensity score matching (PSM) | | | | | | |
| Variables | Before PSM | | | After PSM | | |
|  | Immunocompromised patients (N = 68) | Immunocompetent patients (N = 188) | Standardized difference, % | Immunocompromised patients (N = 43) | Immunocompetent patients (N = 43) | Standardized difference, % |
| Male sex, % | 64.70% | 72.90% | -18% | 74.4% | 76.7% | -5% |
| Age, mean | 56.7 | 55.1 | 1% | 57.2 | 57.6 | 0% |
| Body mass index, mean | 24.4 | 26.4 | -8% | 25.0 | 24.8 | 1% |
| VV ECMO, % | 92.60% | 85.10% | 24% | 88.4% | 81.4% | 20% |
| Interval of MV to ECMO (hours), mean | 128.8 | 103.5 | 0% | 104.5 | 113.9 | 0% |
| Modified Charlson comorbidity index, mean | 4.7 | 3.9 | 9% | 4.4 | 4.5 | -1% |
| Prone position before ECMO, % | 13.20% | 10.10% | 10% | 16.3% | 11.6% | 14% |
| iNO before ECMO, % | 32.40% | 23.90% | 19% | 27.9% | 23.3% | 11% |
| Modified APACHE II score, mean | 22.3 | 21.0 | 2% | 21.3 | 22.9 | -4% |
| Inotropic score, mean | 23.5 | 19.9 | 0% | 20.5 | 21.2 | 0% |
| Dynamic driving pressure (cmH2O) , mean | 16.7 | 18.6 | -4% | 15.9 | 17.7 | -5% |
| Mechanical power (J/min) , mean | 24.5 | 29.9 | -2% | 23.7 | 27.9 | -3% |
| **p* < 0.05  APACHE, Acute Physiology and Chronic Health Evaluation; ARDS, acute respiratory distress syndrome; ECMO, extracorporeal membrane oxygenation; iNO, inhaled nitric oxide; IQR, interquartile range; MV, mechanical ventilation; SD, standard deviation; VV, venovenous | | | | | | |

| **Supplementary Table 6** Baseline characteristics of the patients grouped by the use of the prone position before ECMO | | | |
| --- | --- | --- | --- |
| Variables | Prone  (N = 28) | Non-prone  (N = 228) | *p* value |
| Male sex, n (%) | 22 (78.6) | 159 (69.7) | 0.386 |
| Age, median (IQR) | 59.0 (48.8 – 69.0) | 57.6 (45.5 – 65.5) | 0.489 |
| Body mass index, median (IQR) | 24.8 (23.1 – 27.1) | 25.2 (22.1 – 29.0) | 0.475 |
| VV ECMO, n (%) | 25 (89.3) | 198 (86.8) | 1.000 |
| Interval of MV to ECMO (hours), median (IQR) | 142.5 (31.5 – 312) | 34 (9 – 127) | 0.004* |
| Immunocompromised, n (%) | 9 (32.1) | 59 (25.9) | 0.499 |
| Underlying diseases |  |  |  |
| Charlson comorbidity index, median (IQR) | 5 (3 – 8) | 4 (2 – 6.5) | 0.112 |
| Modified Charlson comorbidity index, median (IQR) | 5 (3 – 6) | 4 (2 – 6) | 0.054 |
| Congestive heart failure, n (%) | 5 (17.9) | 54 (23.7) | 0.636 |
| Hypertension, n (%) | 11 (39.3) | 98 (43.0) | 0.840 |
| Diabetes mellitus, n (%) | 7 (25.0) | 65 (28.5) | 0.825 |
| Coronary artery disease, n (%) | 4 (14.3) | 30 (13.2) | 0.774 |
| Remote stroke, n (%) | 0 | 11 (4.8) | 0.615 |
| Cirrhosis of the liver, n (%) | 2 (7.1) | 10 (4.4) | 0.627 |
| Pre-ECMO dialysis, n (%) | 1 (3.6) | 3 (1.3) | 0.373 |
| Adjunctive treatment |  |  |  |
| Neuromuscular blockers, n (%) | 23 (82.1) | 128 (56.1) | 0.008* |
| iNO before ECMO, n (%) | 19 (67.9) | 48 (21.1) | < 0.001* |
| Initial disease severity |  |  |  |
| Severe ARDS, n (%) | 25 (89.3) | 202 (88.6) | 1.000 |
| APACHE II score, median (IQR) | 26 (21.5 – 30.5) | 21 (15 – 27) | 0.017* |
| Modified APACHE II score, median (IQR) | 25 (20 – 30.3) | 20 (15 – 27) | 0.024* |
| Inotropic score, median (IQR)** | 10.3 (0 – 19.5) | 7.6 (0 – 30) | 0.872 |
| Ventilator setting |  |  |  |
| Dynamic driving pressure (cmH2O), median (IQR) | 17 (12 – 22.3) | 18 (14 – 22) | 0.275 |
| Mechanical power (J/min), median (IQR) | 25.4 (15.5 – 39.3) | 23.9 (16.9 – 33.0) | 0.732 |
| **p* < 0.05  **The inotropic score was calculated as 100 × epinephrine dose (μg/kg/min) + 100 × norepinephrine dose (μg/kg/min) + dopamine dose (μg/kg/min) + dobutamine dose (μg/kg/min)  APACHE, Acute Physiology and Chronic Health Evaluation; ARDS, acute respiratory distress syndrome; ECMO, extracorporeal membrane oxygenation; iNO, inhaled nitric oxide; IQR, interquartile range; MV, mechanical ventilation; SD, standard deviation; VV, venovenous | | | |


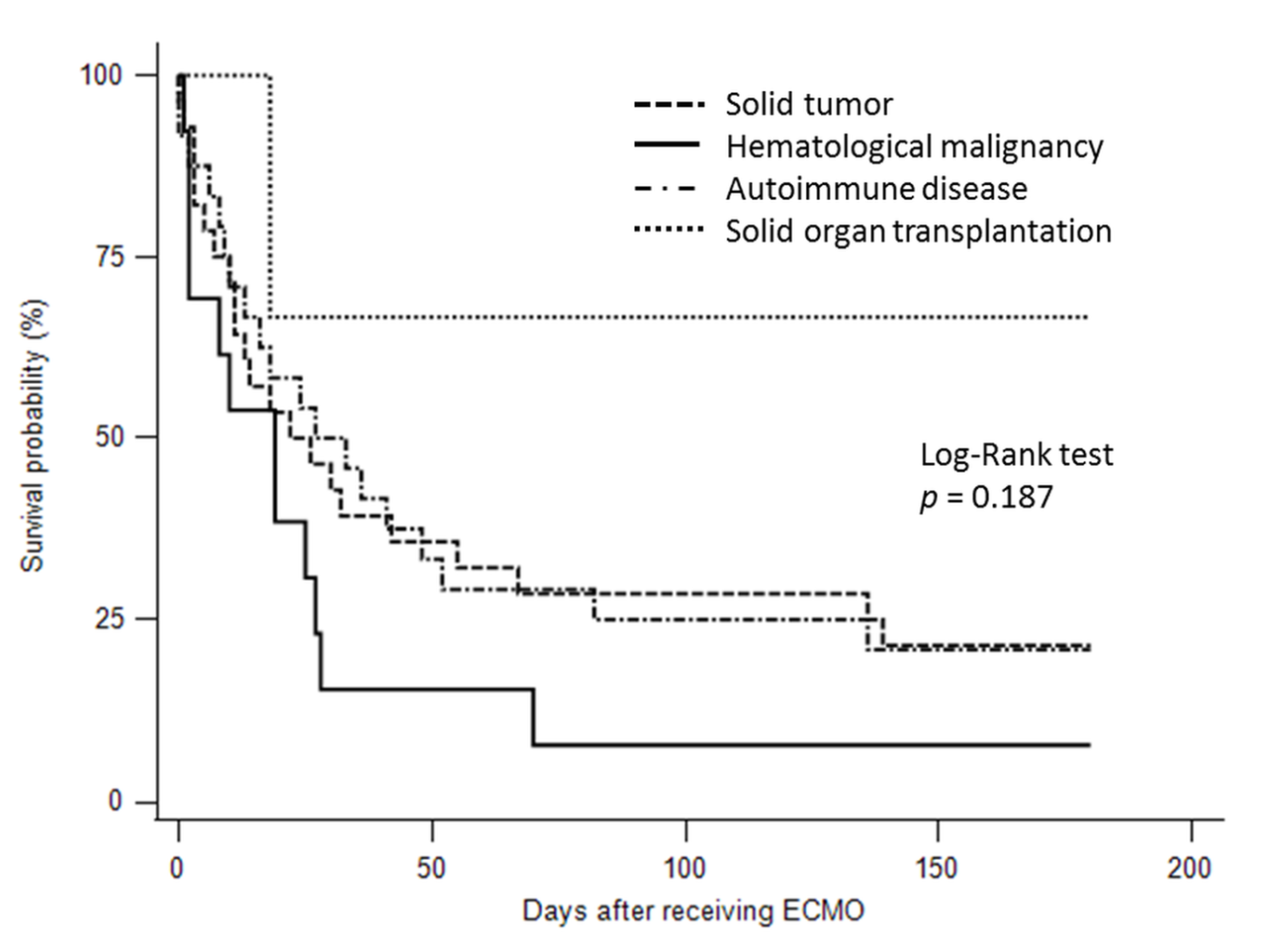


**Supplementary Figure 1** Kaplan-Meier survival curves for 6-month survival in the immunocompromised patients with different causes of immunocompromised status
